# Insight Into Molecular Determinants of T3 vs T4 Recognition From Mutations in Thyroid Hormone Receptor *α* and *β*

**DOI:** 10.1210/jc.2018-02794

**Published:** 2019-02-28

**Authors:** Karn Wejaphikul, Stefan Groeneweg, Yvonne Hilhorst-Hofstee, V Krishna Chatterjee, Robin P Peeters, Marcel E Meima, W Edward Visser

**Affiliations:** 1 Department of Internal Medicine, Erasmus Medical Center, Academic Center for Thyroid Diseases, Rotterdam, Netherlands; 2 Department of Pediatrics, Faculty of Medicine, Chiang Mai University, Chiang Mai, Thailand; 3 Department of Clinical Genetics, Leiden University Medical Center, Leiden, Netherlands; 4 Wellcome-MRC Institute of Metabolic Science, University of Cambridge, Cambridge, United Kingdom

## Abstract

**Context:**

The two major forms of circulating thyroid hormones (THs) are T3 and T4. T3 is regarded as the biologically active hormone because it binds to TH receptors (TRs) with greater affinity than T4. However, it is currently unclear what structural mechanisms underlie this difference in affinity.

**Objective:**

Prompted by the identification of a novel M256T mutation in a resistance to TH (RTH)*α* patient, we investigated Met256 in TR*α*1 and the corresponding residue (Met310) in TR*β*1, residues previously predicted by crystallographic studies in discrimination of T3 vs T4.

**Methods:**

Clinical characterization of the RTH*α* patient and molecular studies (*in silico* protein modeling, radioligand binding, transactivation, and receptor–cofactor studies) were performed.

**Results:**

Structural modeling of the TR*α*1-M256T mutant showed that distortion of the hydrophobic niche to accommodate the outer ring of ligand was more pronounced for T3 than T4, suggesting that this substitution has little impact on the affinity for T4. In agreement with the model, TR*α*1-M256T selectively reduced the affinity for T3. Also, unlike other naturally occurring TR*α* mutations, TR*α*1-M256T had a differential impact on T3- vs T4-dependent transcriptional activation. TR*α*1-M256A and TR*β*1-M310T mutants exhibited similar discordance for T3 vs T4.

**Conclusions:**

Met256-TR*α*1/Met310-TR*β*1 strongly potentiates the affinity of TRs for T3, thereby largely determining that T3 is the bioactive hormone rather than T4. These observations provide insight into the molecular basis for underlying the different affinity of TRs for T3 vs T4, delineating a fundamental principle of TH signaling.

## Teaser

Met256-TR*α*1/Met310-TR*β*1 determine the differential bioactivity of T3 vs T4, providing the molecular basis for the clinical concept that T4 functions as prohormone and T3 as bioactive hormone.

Thyroid hormones (THs) are indispensable for normal growth, development, and metabolism. T3 and T4 are the two major forms of TH. In 1952, it was recognized that T3 has greater biological potency than T4 (1–4). This fundamental discovery led to the clinical concept that T4, despite being the most abundant circulating iodothyronine, functions as a prohormone, with T3 being the biologically active hormone. Since then, this paradigm has remained unchanged, although the molecular and structural mechanisms underlying this have not been investigated in detail.

The genomic actions of THs are exerted through binding to the three functional isoforms of TH receptors (TRs), namely TR*α*1, TR*β*1, and TR*β*2, which are highly homologous but have distinctive expression patterns (5–7). Mutations in TR*α* and TR*β* give rise to clinically distinct syndromes in humans, termed resistance to TH (RTH)*α* and RTH*β*, respectively (8–14). RTH*β* patients commonly present with goiter and tachycardia with abnormal thyroid function tests, including high serum free T3 (FT3) and free T4 (FT4) concentrations with normal or slightly increased TSH concentrations. The clinical phenotype of RTH*α* is distinct from RTH*β* and includes growth retardation, macrocephaly, constipation, intellectual disability, and anemia. In RTH*α*, thyroid function tests are typically characterized by high to high-normal FT3, low to low-normal FT4, low reverse T3, and normal TSH concentrations.

The greater biological activity of T3 vs T4 is explained by differences in affinity for the functional isoforms of TH receptors (TRs). The binding affinity of T4 to the TRs is 10- to 30-fold less compared with T3 (15–17). Previous crystallographic studies revealed that the ligand-binding pocket of TR*β*1 is able to accommodate both T3 and T4, although the helix (H)11-H12 loop is more loosely packed in the presence of T4 than T3 (16). These structural adaptations of TR*β*1, which are required to accommodate the larger T4 molecule, have been attributed to possible steric hindrance of its bulky 5′-iodine moiety with the surrounding amino acids, especially the Met residue located at position 310 in TR*β*1. Although no cocrystallization studies of TR*α* with T4 are available, a similar role for Met256 in TR*α* (the equivalent position of Met310 in TR*β*) has been suggested (18). However, no functional studies to support the relevance of these residues for the differences in affinity for T3 and T4 have been performed.

Therefore, we combined structural modeling and *in vitro* approaches to determine the differential role of these Met residues in T3 vs T4 binding by TRs and characterized a newly identified TR*α*1-M256T and previously published TR*β*1-M310T mutations, which naturally occur in patients with RTH (19–21). We showed that these Met residues are of particular importance for the binding of T3 but not T4. This observation provides the underlying molecular and structural basis for the role of T4 as prohormone and T3 as bioactive hormone in a paradigm for TH physiology and daily clinical practice.

## Materials and Methods

### TR*α*-M256T identification

The TR*α*-M256T mutation in an RTH*α* patient was identified by exome sequencing and was confirmed by Sanger sequencing as previously described (12) after obtaining informed consent. This study was conducted following the Declaration of Helsinki principles and was approved by the Medical Ethical Committee of the Erasmus Medical Center, Rotterdam, Netherlands (MEC-2015-362).

### *In silico* prediction of TR*α*1-M256T function

The TR*α*1-M256T mutation bound to T3 and T4 was modeled into the wild-type (WT) TR*α*1 crystal structure (PDB-ID: 2H77) (22), and the M256T and M256A mutations were introduced using the side-chain substitution tool of the YASARA Structure Software (YASARA Bioscience GmbH, Vienna, Austria) (23) and processed as previously described (24).

### DNA constructs and mutagenesis

The pcDNA3 FLAG-TR*α*1 and TR*β*1 expression vectors containing full-length human TR*α*1 and TR*β*1 with 5′ FLAG tagged (11, 24) and the pCMX VP16-TR*α*1 expression vector containing full-length human TR*α*1 fused with VP16 (25) have been described previously. TR*α*1-M256T, TR*β*1-M310T, as well as the other TR*α*1 mutations (M256A, A263S, D211G, and R384H) were introduced using the QuickChange II Mutagenesis kit (Agilent Technologies, Amstelveen, Netherlands) according to the manufacturer’s protocol. The introduced mutations were confirmed by Sanger sequencing.

### Radioligand competitive binding assays

FLAG-TR*α*1 WT, M256T, and M256A receptor proteins were synthesized using the TnT® T7 Quick Coupled Transcription/Translation System (Promega, Leiden, Netherlands). The affinity for T3 and T4 of the receptors was determined by competitive binding assays as previously described (24) using [^125^I]T3 and [^125^I]T4, respectively. The dissociation constant (Kd) was analyzed by GraphPad Prism program version 5.0 (GraphPad, La Jolla, CA) and shown as the mean ± SEM of three independent experiments performed in duplicate.

### Cell culture and transfection

JEG-3 cells (ECACC Cat. no. 92120308, RRID:CVCL_0363; Sigma-Aldrich, Munich, Germany) were cultured and transfected as previously described (24, 26). Given the absence of 5′-deiodinating activity in this cell type (27), there is no intracellular deiodination of T4 to T3, which allowed us to study the direct effect of T3 and T4 on transactivation. For transcriptional activity assays, WT or mutant receptors were coexpressed with luciferase reporter constructs containing direct repeat TH response elements (DR4-TRE) as well as pMaxGFP as a transfection control. We also coexpressed WT and TR*α*1-M256T in a 1:1 equimolar ratio to determine the effect of the mutant on WT function (*i.e.*, the dominant-negative effect). For receptor–cofactor interaction (two-hybrid) assays, VP16-fused WT or TR*α*1-M256T were coexpressed with a luciferase reporter construct containing the Gal4 binding site (UAStkLuc), together with pSG424 expression vectors containing the Gal4DBD fused to the interacting domains of NCoR1 or SRC1 (11). After transfection for 24 hours, cells were stimulated with 0 to 10,000 nM T3 (Cat. no. T2877; Sigma-Aldrich) or T4 (Cat. no. T2376; Sigma-Aldrich) in DMEM/F12 medium supplemented with 0.1% bovine serum albumin for 24 hours.

### Immunoblotting

The expression of FLAG-tagged and VP16-fused receptors in JEG-3 cells was verified by immunoblotting nuclear extracts as previously described (24, 26). FLAG-tagged TR*α*1 and VP16-TR*α*1 were detected with a 1:1000 dilution of FLAG-M2 (#F1804; Sigma-Aldrich) and VP16 (sc-7545; Santa Cruz Biotechnology, Heidelberg, Germany) antibodies. The Histone 3 protein was detected as loading control with a 1:1000 dilution of a Histone 3 antibody (1B1B2) (#14269 Cell Signaling Technology, Leiden, Netherlands).

### Luciferase assays

Luciferase activity was measured as previously described (12, 24). Data were expressed as percentage maximal response of WT stimulated by T3. EC50, IC50, and maximal response were calculated using GraphPad Prism program version 5.0 (GraphPad, La Jolla, CA). The results are shown as the mean ± SEM of at least three independent experiments performed in triplicate.

### Statistical analysis

Statistical differences of logKd, logIC50, and logEC50 values between groups were analyzed by Student *t* test or one-way ANOVA with Tukey post test. The percentage maximal response of mutants was compared with WT by one-sample *t* test. Statistical significance was considered at *P* < 0.05.

## Results

### Clinical characterization

**Figure 1.**
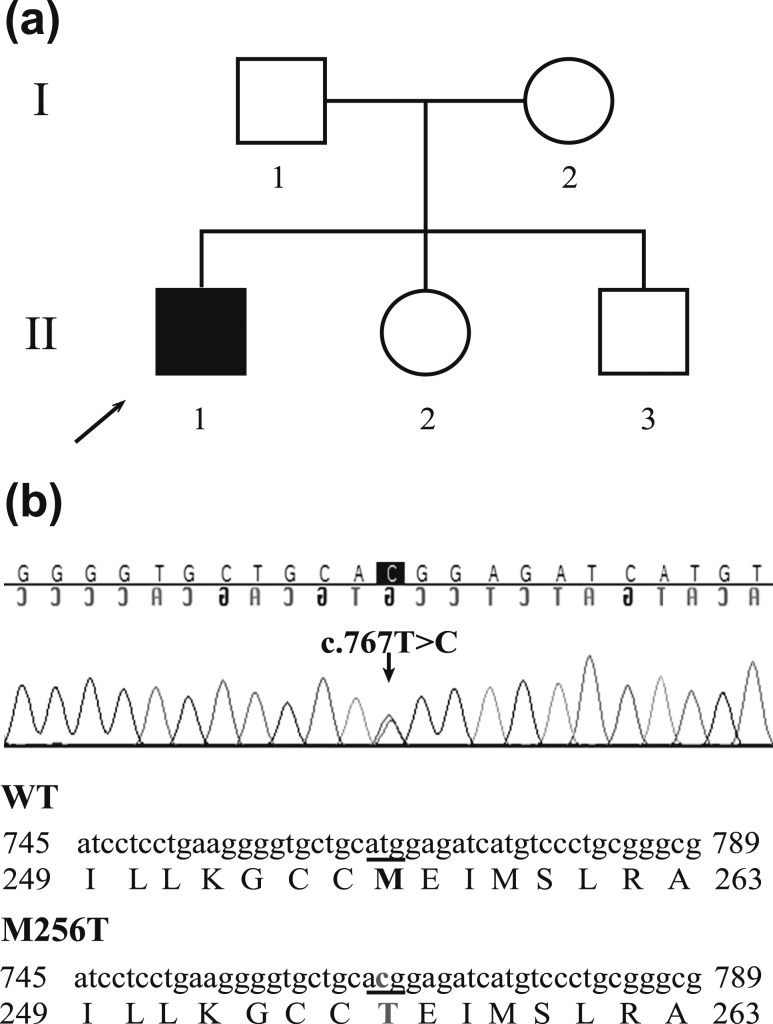
(a) Pedigree chart demonstrating that only the index patient (II.1) has the clinical phenotype of RTH*α*. (b) Sequence analysis of exon 8 of the *THRA* gene shows a *de novo* heterozygous missense mutation (c.767T>C) in the index patient, resulting in a Met to Thr substitution at codon 256 (p.M256T).

A *de novo* heterozygous missense mutation in the *THRA* gene (c.767T>C), resulting in substitution of Thr for Met at codon 256 (p.M256T), was identified in a 19-year-old male patient presenting with features similar to previously reported RTH*α* patients, including disproportionate ischial leg length (sitting height to height ratio +2.5 SD score), mild neurodevelopmental delay, coarse facies, macrocephaly (head circumference 60 cm, +2.5 SD score), and high serum T3/T4 ratio with normal TSH concentrations [FT4, 10.6 pmol/L (normal range, 11 to 25 pmol/L); total T4, 67 nmol/L (normal range, 58 to 128 nmol/L); total T3, 2.9 nmol/L (normal range, 1.4 to 2.5 nmol/L); reverse T3, 0.18 nmol/L (normal range, 0.22 to 0.54 nmol/L); T3/T4 ratio, 0.043 (normal range, 0.01 to 0.03); and TSH, 1.83 mU/L (normal range, 0.4 to 4.3 mU/L)] (Fig. 1). This mutation is not present in public databases (dbSNP, 1000Genome, and Exome Aggregation Consortium).

### Protein modeling

The role of the Met256 in TR*α*1 function and the potential effect of this mutation on the affinity of T3 and T4 were predicted by *in silico* modeling. Given the absence of a T4-bound TR*α* crystal structure, we studied the structural organization of the domains surrounding the outer ring of TH in the available T3- (PDB ID: 1xzx) and T4-liganded (PDB ID: 1y0x) crystal structures of TR*β*1. In line with a previous report (16), we observed that the 5′ position of the outer ring of T3 and T4 is flanked by Ile276 (H3), Met310 and Met313 (H6), His435 (H11), and Phe455 and Phe459 (H12) of TR*β*1. Together, these residues form a niche that allows the accommodation of T4 despite the presence of its bulky 5′-iodine. The same niche is present within the T3-liganded TR*β*1 crystal but is considerably smaller in the absence of the 5′-iodine. Met310 (corresponding to Met256 of TR*α*1) is in the closest structural proximity to the 5′-carbon of the outer ring and forms an extensive network of (hydrophobic) interactions that link H6, H11, and H12.

**Figure 2.**
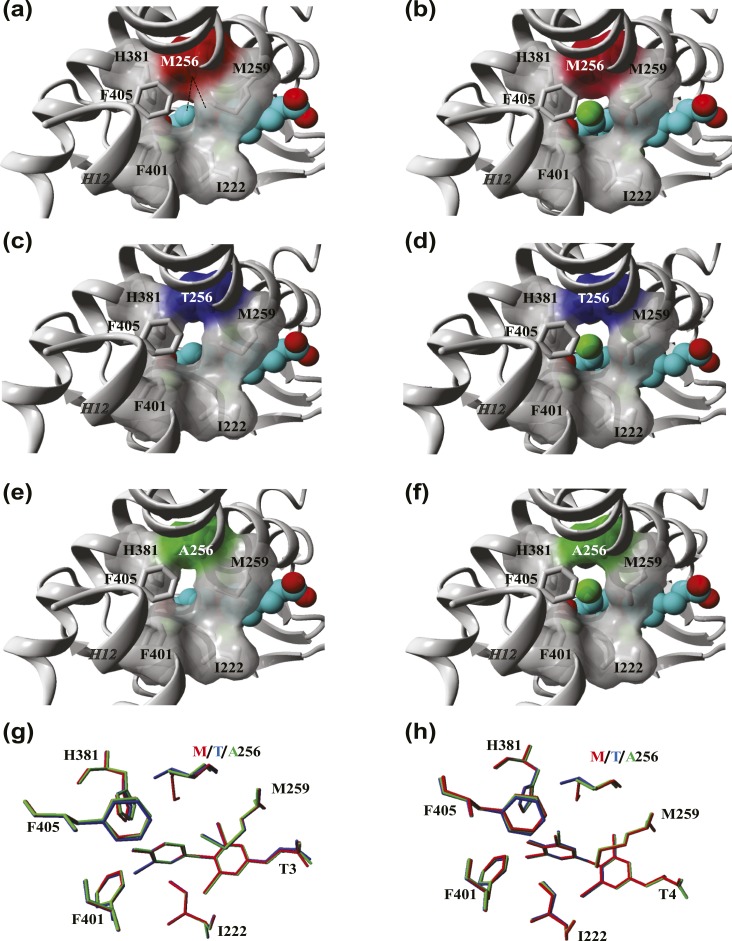
Comparison of the architecture of the TR*α*1 ligand binding pocket in the presence of T3 and T4. (a and b) Close-up view of the ligand-binding pocket of the TR*α*1 crystal structure in complex with (a) T3 (PDB ID: 2h77) and (b) T4. The residue side-chains lining the niche that accommodates the outer ring of T3 and T4 are highlighted, and their molecular surface is shown except for Phe405 for clarity. The 5′-iodine group of T4 is represented by the green ball in the T4-bound TR*α*1 model. The hydrophobic contacts between Met256 and the phenolic outer ring are depicted as dashed lines. (c and d) Structural models of the TR*α*1-M256T mutant in complex with (c) T3 and (d) T4. (e and f) Structural models of the TR*α*1-M256A mutant in complex with (e) T3 and (f) T4. (g and h) Overlay of the structural orientation of the residue side-chains that face the (g) T3 and (h) T4 ligands at the 5′ position in WT (gray), M256T (blue), and M256A (red) mutant TR*α*1 models. All figures were created in YASARA Structure using PovRay imaging software.

We next modeled a T4 molecule into the ligand binding pocket of the available T3-liganded TR*α*1 crystal structure (PDB-ID: 2H77) [Fig. 2(b)]. Compared with the T3-liganded TR*α*1 structure [Fig. 2(a)], a slight outward shift of H11 and H12 was observed in the T4-liganded model, which was accompanied by reorientation of side-chains of residues surrounding the 5′-iodine. This resulted in a loss of the direct hydrophobic interactions between Met256 and the outer ring and a less tightly packed structural organization of the ligand binding pocket. These changes were similar to those observed in the corresponding TR*β*1 crystal structures, validating the accuracy of the modeling procedure.

We subsequently modeled the M256T (shortening of side-chain, hydrophilic moiety) mutant in both T3- and T4-bound TR*α*1 structures and analyzed the impact on the conformation of the ligand binding domain and direct substrate interactions [Fig. 2(c) and 2(d)]. The artificial M256A mutant was also modeled to reduce the side-chain length while maintaining the hydrophobic property of the residue [Fig. 2(e) and 2(f)]. Due to shortening of side-chain length in both mutants, direct hydrophobic interaction with the outer ring of T3 was lost [Fig. 2(c) and 2(e)]. Moreover, both mutants enlarged the niche surrounding the 5′ position of T3 due to reorientation of various residue side-chains in H11 and H12 and the subsequent outward shift of these helices. As a result, the niche adopts a structural configuration that resembles the WT receptor in the T4-bound state. These changes were more pronounced for the M256T than the M256A, exemplified by the degree of reorientation of His381, which was previously implicated to interact with the phenolhydroxyl group of T3 (18) [Fig. 2(g)]. In the case of T4, both mutations had little effect on structural organization [Fig. 2(d), 2(f), and 2(h)]. Based on these *in silico* predictions, we hypothesized that both substitutions would have a greater impact on T3 than on T4 binding and action.

### Functional studies

**Figure 3.**
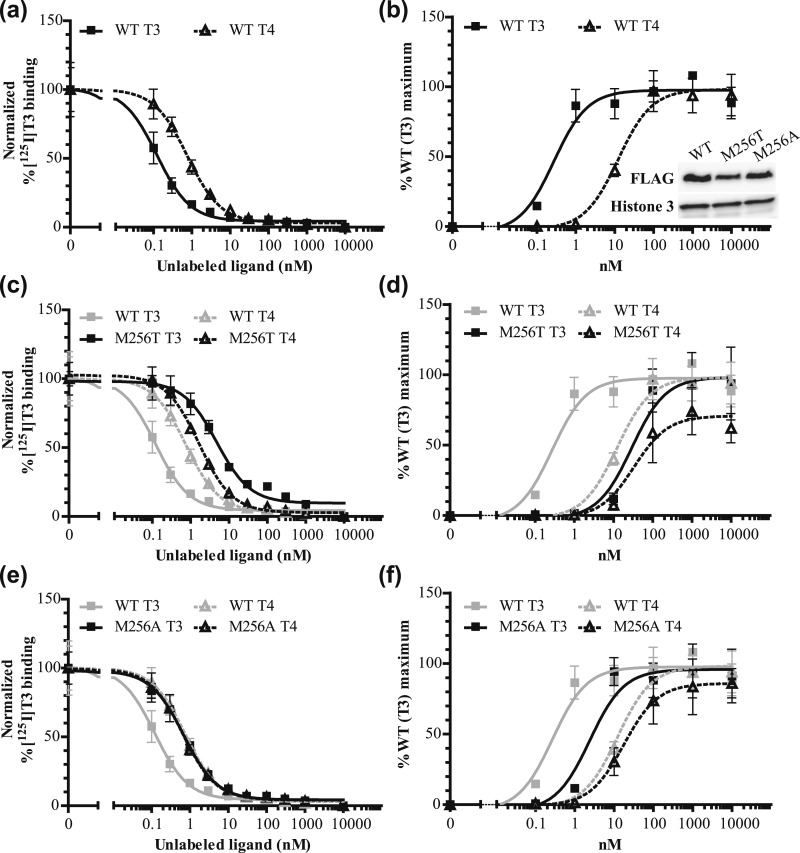
(a, c, e) [^125^I]T3 dissociation curves showing that compared with (a) WT, (c) the TR*α*1-M256T mutation, and (e) TR*α*1-M256A mutation reduces the affinity for T3 (solid line) more than for T4 (dashed line) (mean ± SEM of three experiments for WT and M256T and two experiments for M256A performed in duplicate). (b, d, f) The TR*α*1-M256T and TR*α*1-M256A mutations also had a larger effect on T3- than on T4-dependent transcriptional activation (mean ±SEM of three experiments performed in triplicate). The effect of the Ala substitution on the ligand binding affinity and the transcriptional activity of TR*α*1 was less than the effect of the Thr substitution. The insert in (b) shows immunoblots confirming an equal expression of WT, M256T, and M256A FLAG-tagged TR*α*1 and Histone 3 as a loading control in the nuclear fraction of JEG-3 cells.

We performed *in vitro* studies to test this hypothesis. In line with previous literature (15–17), competitive binding assays showed that the affinity for T4 of WT TR*α*1 was approximately sevenfold lower than for T3, indicated by a higher Kd of T4 than T3 [Fig. 3(a); Table 1]. The TR*α*1-M256T mutant showed a ∼40-fold lower T3 binding affinity than WT, whereas T4 affinity was unchanged [Fig. 3(c); Table 1]. Also, the binding affinity of the TR*α*1-M256A mutant for T3 was selectively reduced (approximately sixfold) [Fig. 3(e); Table 1].

**Table 1.** Summary of the Results of Competitive Binding, Transcriptional Activity, and Protein-Protein Interaction Assays of WT, TR*α*1-M256T, and TR*α*1-M256A Mutants

	T3 Stimulation			T4 Stimulation		
	WT	M256T	M256A	WT	M256T	M256A
LogKd	−0.91 ± 0.08	0.71 ± 0.10^*a*^	−0.16 ± 0.34^*b*^^,^^*c*^	−0.09 ± 0.10	0.22 ± 0.05	−0.18 ± 0.02
Kd(nM)	0.12	5.14	0.69	0.81	1.67	0.66
LogEC50-DR4	−0.60 ± 0.10	1.51 ± 0.16^*a*^	0.51 ± 0.08^*b*^^,^^*d*^	1.16 ± 0.07	1.67 ± 0.11	1.44 ± 0.25
EC50(nM)	0.25	32.3	3.26	14.5	46.6	27.2
LogIC50-NCoR1	−1.26 ± 0.04	0.69 ± 0.18^*a*^	—	0.02 ± 0.06	0.82 ± 0.14^*b*^	—
IC50(nM)	0.06	4.87	—	1.05	6.64	—
LogEC50-SRC1	−0.76 ± 0.05	1.19 ± 0.07^*a*^	—	0.42 ± 0.07	1.16 ± 0.08^*b*^	—
EC50(nM)	0.17	15.5	—	2.65	14.6	—

Data are presented as mean ± SEM (one-way ANOVA with Tukey posttest).

^a^ *P* < 0.001 for WT vs mutant.

^b^ *P* < 0.01.

^c^ *P* < 0.001 for M256T vs M256A.

^d^ *P* < 0.01.

**Figure 4.**
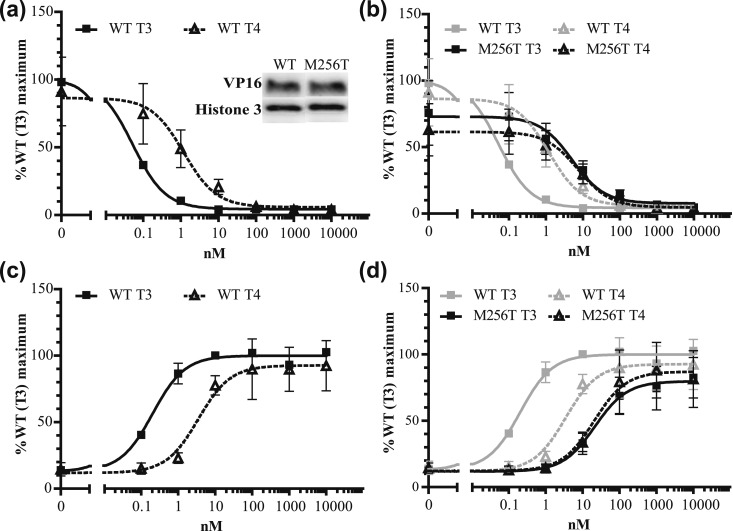
The TR*α*1-M256T mutation had a larger effect on T3- than on T4-dependent (a and b) GAL4-NCoR1 dissociation and (c and d) GAL4-SRC1 association (mean ± SEM of at least three experiments performed in triplicate). The insert in (a) shows immunoblots confirming an equal expression of WT and M256T VP16 TR*α*1 fusion proteins and Histone 3 as loading control in the nuclear fraction of JEG-3 cells.

To evaluate the impact of both mutations on the transcriptional activity, WT and mutant receptors were cotransfected with a reporter construct in which luciferase expression is under control of a TH response element into JEG-3 cells with increasing concentrations of T3 or T4. Equal expression of WT and both mutants was confirmed by immunoblotting nuclear extracts with anti-FLAG antibodies [Fig. 3(b)]. In line with the binding assays and previous studies (16, 17), the transcriptional activation assay showed that the EC50 of WT TR*α*1 induced by T4 was ∼60-fold higher than that induced by T3 [Fig. 3(b); Table 1]. The EC50 of TR*α*1-M256T was 100-fold higher for T3 but was unchanged for T4 compared with WT [Fig. 3(d); Table 1]. The TR*α*1-M256A also selectively reduced transcriptional activity induced by T3 [Fig. 3(f); Table 1]. The transcriptional activity was also reduced when WT and TR*α*1-M256T were coexpressed compared with WT expressed alone, suggesting a dominant-negative effect of this mutant (data not shown). In mammalian two-hybrid assays compared with WT, the TR*α*1-M256T mutant also affected ligand-dependent interactions with the corepressor NCoR1 (fold increase IC50: ∼80-fold for T3 and approximately sixfold for T4) and the coactivator SRC1 (fold increase EC50: ∼90-fold for T3 and approximately sixfold for T4) [Fig. 4(a)–4(d); Table 1]. Together, our results indicate that the mutations located at the Met256 of TR*α*1 have a differential impact on the binding and activation of the receptor by T4 vs T3.

**Figure 5.**
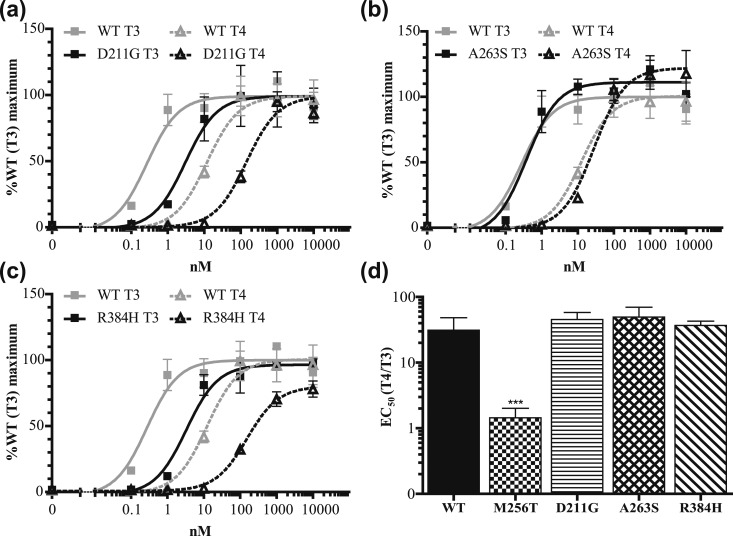
(a–c) The T4-induced transcriptional activity of three TR*α*1 mutations identified in RTH*α* patients is lower than that is induced by T3, which is similar to WT [Fig. 2(d)] (mean ± SEM of three experiments performed in triplicate). (d) The EC50 of T4 is ∼30- to 50-fold higher than the EC50 of T3, except for TR*α*1-M256T. ***P < 0.001 (one-way ANOVA with Tukey post test).

**Figure 6.**
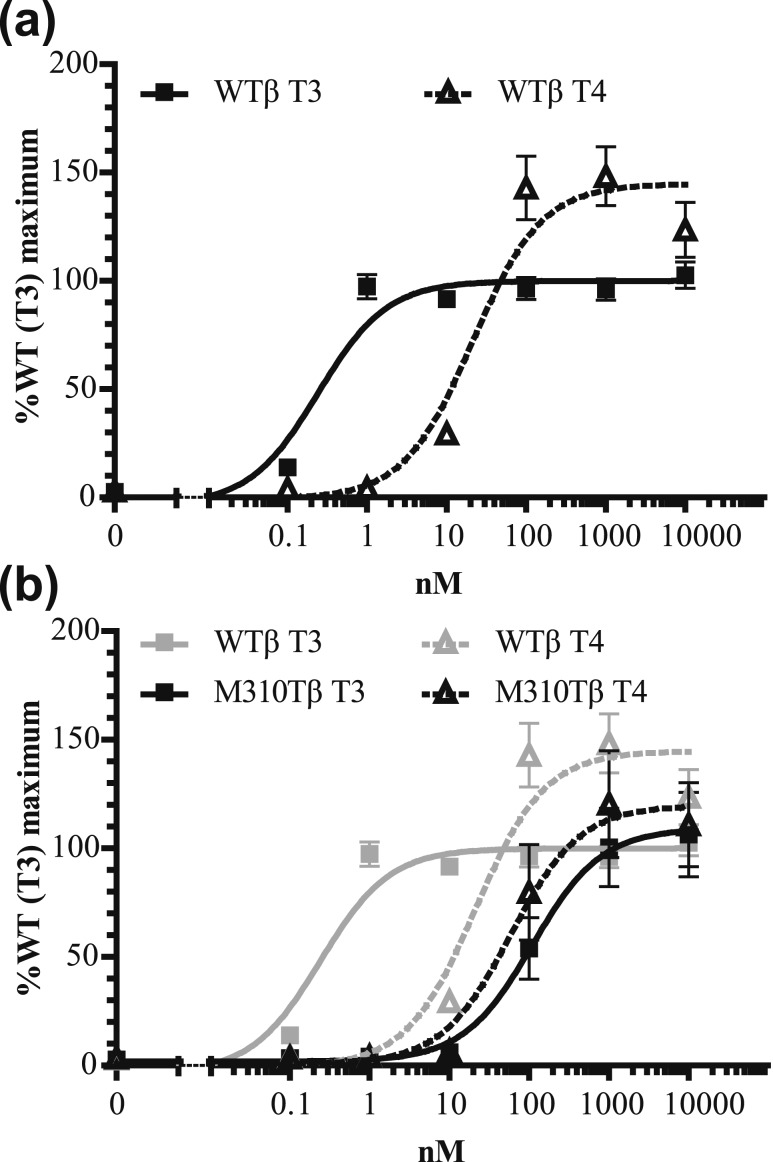
The T3- and T4-induced transcriptional activity of (a) WT and (b) TR*β*1-M310T in JEG-3 cells shows that the TR*β*1-M310T mutation affects T3- more than T4-dependent transcriptional activation (mean ± SEM of four experiments performed in triplicate), which is in line with the results of TR*α*1-M256T [Fig. 3(d)].

We next investigated if this T3 vs T4 difference is present in other TR*α* mutants located outside the niche surrounding the 5′-iodine position. However, these naturally occurring mutations (D211G, A263S, and R384H) had a similar impact on T3- and T4-induced transactivation, and, as for WT TR*α*, the EC50 values for T4 exceeded those for T3 by ∼30- to 50-fold [Fig. 5(a)–5(c)]. These transcriptional activation profiles were in contrast to the M256T mutant [Fig. 5(d)], strongly indicating that only this mutant has a predominant impact on T3 affinity. To extend our findings to TR*β*, we studied the transcriptional activity of a corresponding mutation in TR*β*1 (TR*β*1-M310T). The EC50 of WT TR*β*1 induced by T4 was ∼70-fold higher than that induced by T3 [Fig. 6(a)], which was similar to WT TR*α*1. The T3-induced transcriptional response of TR*β*-M310T was greatly reduced, which contrasted with the T4-induced transcriptional activity (fold increase EC50: ∼350-fold for T3 and approximately threefold for T4) [Fig. 6(b)].

## Discussion

Although the notion of T4 and T3 being the precursor and active hormones, respectively, is widely recognized in the clinical and scientific communities, the molecular and structural basis of this dogma has received little attention. In this study, we highlight the crucial role of residue Met256 of TR*α*1 and Met310 of TR*β*1 in determining the differential bioactivity of T3 vs T4 using a novel mutant (TR*α*1-M256T) identified in an RTH*α* patient and a mutant at the corresponding position (TR*β*1-M310T) identified in RTH*β* patients (19–21). In contrast to WT TR*α* or TR*β* and mutations involving other residues, mutations at these Met residues selectively affected binding and transactivation of TR by T3. These observations emphasize the key role of these residues in designating T4 as the prohormone and T3 as the major bioactive hormone.

In line with previous reports (15–17), our results showed that T3 has a higher binding affinity for WT TR*α*1 and stimulates receptor activity with a higher potency than T4. Previous structural studies in TR*β*1 have suggested that the lower affinity for T4 is caused by decreased packing of the ligand binding domain in the presence of T4 vs T3, which particularly allows oscillation of H12 between liganded and unliganded states, resulting in a higher ligand dissociation rate (16). Here, we extend these observations by showing that the ligand binding domain of T3-liganded TR has a similar decrease in packing as observed in T4-liganded WT receptors upon substitution of Met256 in TR*α*1 or Met310 in TR*β*1 by Thr. In contrast, these substitutions hardly changed the predicted structure of T4-liganded mutant receptors. Based on these models, we postulated that the extensive (hydrophobic) interactions of Met with surrounding residues are key in stabilizing interhelical interactions (*e.g.*, between H6, H11, and H12), which facilitate the tight packing of the ligand binding domain as observed in T3-liganded receptors. Moreover, we observed a direct interaction between Met and the 5′ position of the outer ring of T3, which was not formed with T4. This suggests that Met256 in TR*α*1 and Met310 in TR*β*1 have a critical role in achieving optimal folding and enthalpy in T3-liganded receptors, whereas their role in T4 binding is of less importance.

This *in silico* prediction was confirmed by *in vitro* studies indicating that TR*α*1-M256T selectively affected binding affinity for T3 as well as cofactor interactions and transcriptional activity of the T3-stimulated receptor. These properties seemed specific for the M256T mutant because the transactivation potency of T3 and T4 with TR*α* mutants identified in other RTH*α* patients [D211G (26), A263S, and R384H (28)] was affected equally. Additional testing of the naturally occurring mutation at the corresponding residue in the TR*β*1 (M310T) (19–21) further substantiated the specificity of the findings.

Thr substitution at position 256 in TR*α*1 or 310 in TR*β*1 not only alters the binding space but also affects the hydrophobicity of the ligand-binding pocket. Therefore, we tested the artificial TR*α*1-M256A mutant, which reduces the size of the side-chain but maintains the hydrophobic property of the ligand-binding pocket. Indeed, functional studies showed that TR*α*1-M256A also selectively impairs T3 binding affinity and T3-induced transcriptional activity, whereas T4 binding and activity are maintained. Although the effect of the TR*α*1-M256T mutation in our functional and structural models was slightly greater than that of TR*α*1-M256A, these findings support the notion that loss of the specific properties of Met, rather than the unfavorable impact of the hydrophilic moiety of Thr on the hydrophobic environment, are mainly responsible for the differential impact on T3 vs T4 signaling. Based on our studies and on a previous report (16), we propose that Met256 in TR*α*1 and Met310 in TR*β*1 are crucial residues that determine specific affinity for T3 vs T4. Thr and Ala substitution at these Met positions significantly affected the hydrophobic interactions with T3 and altered the niche accommodating the outer ring of T3 to a “T4-bound” configuration, both resulting in a reduced binding affinity of the mutants for T3. In contrast, because the ligand binding domain of T4-liganded receptors already exhibit looser packing without direct interaction(s) between Met and the T4 molecule, mutations in the Met residue are better tolerated.

No unique phenotype was discernible in the newly identified M256T RTH*α* patient when compared with other cases of RTH*α* harboring missense mutations in the *THRA* gene (25, 26, 28–30) or in patients carrying TR*β*-M310T (19–21) when compared with other RTH*β* cases reported in the literature. These findings indicate that, although mutations at Met256-TR*α*1/Met310-TR*β*1 residues preserve T4 binding to mutant receptor proteins, this property is not sufficient to prevent patients from developing features of RTH, implying that the phenotype of RTH is linked primarily to defective T3 rather than T4 binding by mutant TRs.

This study provides *in vitro* evidence for the importance of Met256 in TR*α*1 and Met310 in TR*β*1 in ligand recognition. Our studies highlight the relevance of this Met residue in TRs for discrimination between T3 and T4, providing the molecular basis for the role of T4 as prohormone and T3 as bioactive hormone.

## Abbreviations:

FT3: free T3
FT4: free T4
RTH: resistance to thyroid hormone
TH: thyroid hormone
TR: thyroid hormone receptor
WT: wild type
